# Supplementary material for: The interactome of intact mitochondria by cross-linking mass spectrometry provides evidence for coexisting respiratory supercomplexes
Source: Mol Cell Proteomics. 2017 Dec 8;17(2):216–32. doi: 10.1074/mcp.RA117.000470 (PMC5795388; doi:10.1074/mcp.RA117.000470)
Supplement: Supplemental Data [file supp_RA117.000470_133922_0_supp_23978_fzffwf.zip › spectra_annotation/mito_DR_spectra_annotation/14-1-4-1-16-1.pdf]

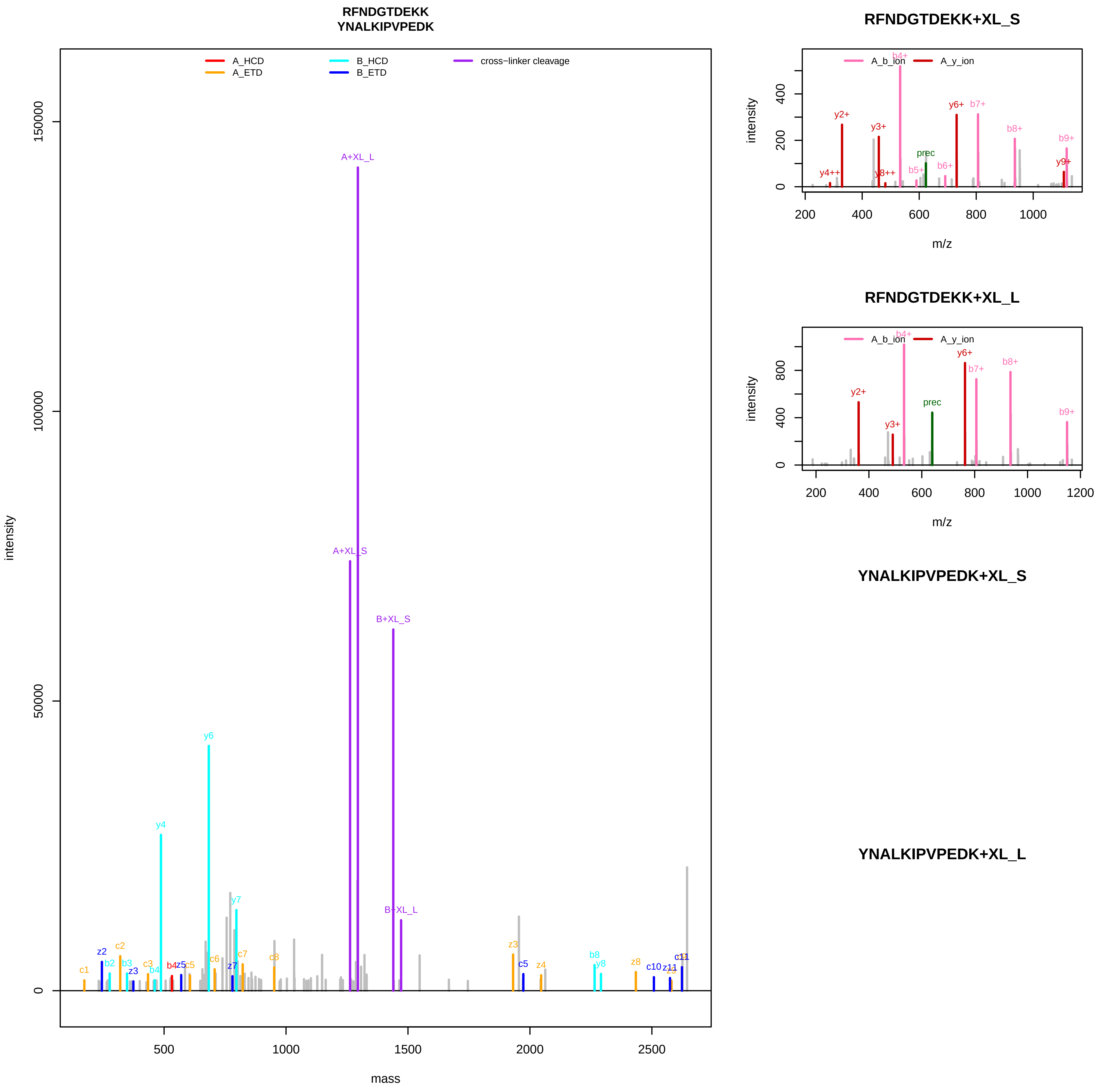

RFNDGTDEKK
YNALKIPVPEDK
A_HCD
A_ETD
B_HCD
B_ETD
cross−linker cleavage
A+XL_L
A+XL_S
B+XL_S
B+XL_L
y6
y4
y7
y8
b8
intensity
mass
150000
100000
50000
0
500
1000
1500
2000
2500
RFNDGTDEKK+XL_S
A_b_ion
A_y_ion
b4+
y2+
y3+
y6+
b7+
b8+
b9+
y9+
prec
b5+
b6+
y4++
y8++
m/z
RFNDGTDEKK+XL_L
YNALKIPVPEDK+XL_S
YNALKIPVPEDK+XL_L
